# Surgical treatments for acute unstable acromioclavicular joint dislocations

**DOI:** 10.1051/sicotj/2022038

**Published:** 2022-09-07

**Authors:** Georgios Saraglis, Aditya Prinja, Kendrick To, Wasim Khan, Jagwant Singh

**Affiliations:** 1 Department of Trauma and Orthopaedics, Lewisham and Greenwich NHS Trust London SE13 6LH UK; 2 Upper Limb Unit, Wrightington Hospital Lancashire UK; 3 Division of Trauma and Orthopaedic Surgery, Department of Surgery, University of Cambridge Cambridge CB2 0QQ UK

**Keywords:** Acromioclavicular joint dislocation, Acromioclavicular joint reconstruction, Hook plate fixation, Coracoclavicular ligament reconstruction

## Abstract

*Introduction*: Surgical treatment is usually recommended for acute, high-grade acromioclavicular joint (ACJ) injuries. A wide variety of surgical techniques exist, and the literature does not strongly support one over the other. In this literature review, we describe and compare the results of different surgical treatments for the management of acute unstable ACJ dislocation and aim to guide surgeons on optimal treatment. *Materials and methods*: A literature review was performed by searching PubMed, Medline, Cochrane, and Embase databases. Seventeen studies met the inclusion criteria and were analyzed. Only studies with comparative data were included. The clinical and radiological outcomes of these studies were reviewed. *Results*: Seventeen studies were included in this literature review. We found no difference in outcomes between open and arthroscopic procedures. Coracoclavicular ligament (CCL) reconstruction techniques provide better results than the more rigid hook plate fixation. There is no evidence that biologic repair with tendon graft is superior to synthetic grafts. Furthermore, an autograft is not shown to be better than an allograft. Rigid fixation between the clavicle and coracoid and the non-anatomic Weaver-Dunn technique appears less popular in recent literature. The hook plate is associated with subacromial osteolysis, acromial erosion, and the morbidity of a secondary procedure. *Discussion*: There is a recent increase in publications on the reconstruction of the ACJ after injury, with new techniques focusing on the anatomic reconstruction of the CCLs aiming to restore both vertical and horizontal plane stability of the ACJ using synthetic/biological grafts. Despite the plethora of new techniques introduced, meaningful comparisons are difficult to draw due to the heterogeneity of the treatments used and the outcome measure used to assess the results.

## Introduction

Acromioclavicular joint (ACJ) dislocation is responsible for approximately 9% of shoulder girdle injuries [1]. The most commonly used classification by Rockwood is a radiographic description of the injury from type I to VI [2]. Type I and II injuries can be treated non-operatively, whereas types IV–VI usually necessitate surgical intervention due to the severity of the injury [3, 4]. However, controversy exists regarding the optimal treatment for type III injuries [5].

When surgery is required, many techniques exist, including reduction and fixation of the ACJ and reconstruction of the coracoclavicular ligaments (CCLs) [6]. ACJ fixation can be achieved using screws, wires, or the hook plate. Complications of these techniques include a failure to adequately maintain ACJ reduction, fractures of the coracoid process, or osteolysis of the lateral end of the clavicle. Hook plate fixation also carries the morbidity of requiring an additional procedure to remove the metalwork [7]. The modified Weaver-Dunn procedure was historically the most commonly used method for reconstruction of the CCL [8] and involves the transfer of the coracoacromial ligament together with a small piece of bone from the acromion to the lateral end of the clavicle.

The congruency of the ACJ strongly depends on both static and dynamic stabilizers [9]. Static stabilizers include the joint capsule and the AC ligaments that reinforce it as well as the CCLs. As the importance of the ligaments in maintaining ACJ stability has become better understood, techniques have been developed to restore or reconstruct the CCLs [10–13]. These include the use of biologic tendon autograft and allograft as well as synthetic materials and single or double bundle techniques [14–16]. Early results of these techniques have been promising, with good clinical outcomes. This literature review aims to present the results of the most recent surgical techniques in managing acute ACJ dislocations. The comparison of open versus arthroscopic techniques, hook plate fixation versus ligament reconstruction, the comparison of different CC ligament reconstruction techniques, different graft options available (synthetic, biological, autograft, allograft), and the comparison of combined techniques we aimed to guide surgeons regarding the optimal treatment option.

## Materials and methods

### Literature Search

Two independent reviewers (GS and JS) on PubMed, Medline, Embase, and the Cochrane Library performed a search of the literature to identify relevant publications. The key search terms used were (“acromioclavicular” OR “coracoclavicular”) AND (“reconstruction” OR “repair”); these operators were adapted according to the database. The literature search was conducted using an advanced search with combinations of these keywords. Each reviewer assessed the studies and applied the inclusion and exclusion criteria during study selection.

### Criteria of inclusion

English language studies that provided data on outcomes of acute unstable ACJ dislocations managed operatively with the reconstructive methods mentioned were included. A study was included if it referred to the comparison between techniques of any of the reconstructive methods in question. Letters, comments, case series with no comparative techniques, case reports, cadaveric studies, biomechanical studies, systematic reviews, and studies involving non-human subjects were excluded. Non-English articles with English abstracts with sufficient detail on methodology and outcomes were included. There was no exclusion based on operative conditions, outcomes of surgery, or length of follow-up.

### Study selection

**Figure 1:**
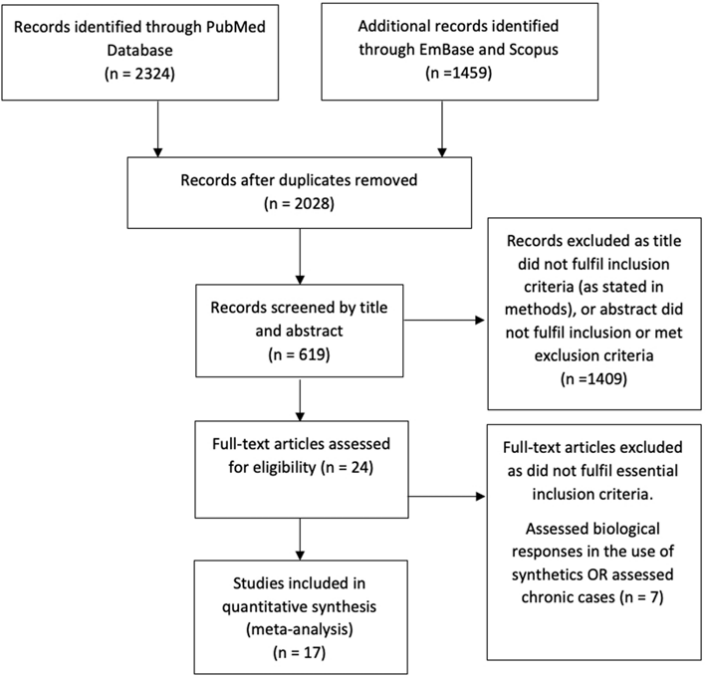
Summary of exclusion criteria.

A total of 2028 records were initially identified (after the removal of duplicates) through the database search. One thousand four hundred nine articles were excluded per the above criteria. After a full-text review of the remaining 24 studies, 7 further studies were excluded, as these related to the biological responses to synthetic materials used for reconstruction, not the clinical outcomes, or assessed the management of chronic cases. Reasons for exclusion are summarized in Figure 1. The remaining 17 studies were included in the literature review, and data from these studies are included in Table 1.

**Table 1:** Studies included.

Study	Surgical technique	Number of patients	Mean age (years)	Mean follow-up	Post-operative assessment scores	Evaluation of CC distance	Study Design/Level of Evidence	Complications
Hou et al. [40]	Allogenous semitendinosus graft/single tunnel	11	37	16 months	Percentage of good to excellent outcomes: 18%	Not reported	Retrospective study/III	Coracoid fracture: 1 case
	Allogenous semitendinosus graft/two tunnel	10	42		Percentage of good to excellent outcomes: 70%			Wound infection: 2 cases
Kumar et al. [34]	Modified Weaver-Dunn procedure	31	42	40 months	Mean OSS pre op: 28 ± 11	Not reported	Case series/IV	Failure: 3 cases
					OSS post op: 42 ± 10			Superficial wound infection: 3 cases
	Surgilig technique	24			Mean OSS pre op: 26 ± 9			Implant failure: 1 case
					OSS post op: 45 ± 7			Superficial wound infection: 4 cases
Yoon et al. [22]	Hook plate	24	38.8 ± 14.2	16 ± 12.8 months	VAS: 1.6 ± 1.5	Pre op: 215.7 ± 50.9%	Case series/IV	No cases of AC joint subluxation
					CS: 90.2 ± 9.9	Post op: 106.1 ± 10.2%		Acromion erosion: 9 cases
	CC ligament reconstruction/synthetic ligament	18	42.2 ± 12.3	17.4 ± 4.3 months	VAS: 1.3 ± 1.3	Pre op: 239.9 ± 59.2%		AC joint subluxation: 6 cases
					CS: 89.2 ± 3.5	Post op: 133.6 ± 36.7%		
Li et al. [19]	Arthroscopic CC reconstruction/synthetic ligament	32	40.3	29.6 ± 6 months	ASES: 96 ± 5.1	Loss of reduction: 1/32 cases	Retrospective Comparative study/III	Weaver Dunn group: higher rate of loss of reduction
					UCLA: 34.2 ± 1.5			
	Open modified Weaver Dunn	31			ASES: 94.5	Loss of reduction: 7/31 cases		
					UCLA: 33.7 ± 1.4			
Tang et al. [39]	Double Endobutton	31	No significant difference in age between the two groups	11–35 months	CMS: no significant difference between groups (*P* > 0.05)	Not reported	Case series/IV	Loss of CC reduction: 1 case
	Suture anchor + Endobutton plate	25			Karlsson grading no significant difference between 2 groups (*Z* = −0.628, *P* = 0.530)			Ectopic ossification: 6 cases(both techniques)
Barth et al. [41]	Double endobutton arthroscopic technique in 93% of cases.	116	37	12 months	CMS: >85/100	Pre op: 214%(vertical)	Non-randomized comparative study/II	Early loosening: 3 cases
					QuickDASH functional disability <10 in 75% of patients	Pre op: 4 mm (horizontal)		Surgical infection: 2 cases
	Additional acromioclavicular stabilization of the AC joint in 50% of cases.					Post op: 128% (vertical)		Reflex sympathetic dystrophy: 7 patients
						Post op: 0 mm (horizontal)		Distal clavicle osteolysis: 1 case
								Device impingement:5 cases
								Coracoid fracture: 1 case
Cisneros et al. [18]	Arthroscopic CC fixation/synthetic ligament	20	36	>24 months	Global satisfaction: 8.85 ± 0. 93	Not reported	Retrospective Comparative/III	Implant failure: 1 case
					VAS score: 0.40 ± 0.50			Surgical wound granulomas; 2 cases
								Scapular dyskinisis: in 15%
	Hook plate	11	41		Global satisfaction: 8.00 ± 1.18, *P* = 0.035			Surgical site infection: 1 case
					VAS score 1.45 ± 1.51, *P* = 0.007			Inability of metalwork removal: 1 case
								Scapular dyskinesis: in 18.18%
Vrgoc et al. [12]	Open Reduction + K-wires + Fiber-Tape	10	41.6	At least 12 months	No significant statistical difference between techniques	Not reported	Case series/IV	Not specifically reported
	Arthroscopic fixation/Tight-Rope	6	37.8					
Yin F et al. [36]	Autologous plantaris graft +hook plate	15	No difference between age groups	21.4 months	CMS: no difference between techniques	CC distance: similar results between the two techniques	Case series/IV	Not specifically reported
	Allogenic tendon + hook plate	16			VAS: no difference between techniques			
					ROM: no difference between techniques			
Faggiani et al. [20]	Mini open AC repair/MINAR system	8	36.94	13 months	CMS: 89.61	Not reported	Retrospective cohort study/II	Not specifically reported
					OMS: 46.13			
					SST: 11.38			
	Dog Bone arthroscopic technique	8			CMS: 92.6			
					OMS: 46.25			
					SST: 11.75			
Metzlaff et al. [24]	Mini open AC repair (MINAR)/synthetic ligament	44	36.2	32 months	CMS: no difference between techniques	Not reported	Retrospective Comparative/III	Periarticular ossification: 11 cases
					Taft:no difference between techniques			Periarticular ossification: 8 cases
	Hook plate	44			ACJI: no difference between techniques			
Kocaoglu et al. [35]	CC reconstruction/autogenic palmaris Longus graft/GraftRope system	16	39.7	44.9 months	ASES: superior in patients with CC reconstruction	Comparison to the uninjured side: mean 1.1 mm	Retrospective Cohort study/II	Loss of reduction in both groups, higher in the Weaver Dunn group
	Modified Weaver Dunn + Tightrope	16			CMS: superior in patients with CC reconstruction	Comparison to the uninjured side: mean 3.3 mm		
Yin J et al. [37]	Hook plate + double tunnel CC reconstruction/conjoint tendon graft	25	46	19.9 months	CS: 89.5	Increase in the CC distance after the removal of the hook plate by 25–100%	Case-control study/III	Pain and swelling at the site of tendon harvest
					ASES: 92.6			
					VAS: 2.5			
	Hook plate	26	44.5	21–27 months	CS: 79.3			Acromion erosion: 46%
					ASES: 82.3			Subacromial impingement: 23%
					VAS: 4.1			
Wang et al [23]	CC and AC reconstruction/allogenic tendon	8	49	>48 months	CMS: 94.4	Not reported	Case series/IV	No complications noted
					UCLA score: 33.5			
	Hook plate fixation	8	41.3		CMS: 93.8			
					UCLA score: 34.1			
Stein et al [21]	Hook plate	27	37.7	24 months	Taft: 9.4 ± 1.7	Equal loss of reduction CC distance in 24 months	Cohort study/II	No complications noted
					CS: 90.2 ± 7.8			
	Arthroscopic double-button	29	34.2		Taft: 10.9 ± 1.1			
					CS: 95.3 ± 4.4			
Chang et al [38]	Hook plate	26	50	11 months	VAS: 4.5 ± 2.3	CC distance similar in both groups	Therapeutic study/II	Subacromial osteolysis: less in the combined technique group.
					UCLA: 19.9 ± 4.9			
					ASES: 51.9 ± 17.8			
	Hook plate + CC tape augmentation	21	44	9.5 months	VAS: 2.3 ± 1.4			
					UCLA: 27.2 ± 4.0			
					ASES: 73.8 ± 13.1			
Chernchuijit et al [27]	Arthroscopic AC fixation/suture button	29	No difference between age groups	>18 months	SAC: 8	No difference in the CC distance	Retrospective Cohort study/II	Loss of reduction: >50% of patients in the fixation group
					Nottingham: 10			
	Arthroscopic anatomic AC reconstruction/suture tape	29			SAC: 20			
					Nottingham: 19			

### Data extraction

Data collected included the duration of the study, the type of study, number of patients, age, the surgical procedure involved, the patient reported outcome scores, and radiological outcomes. The patient-reported outcomes measures included patient satisfaction, visual analog scale (VAS), Nottingham shoulder score, Constant-Murley score, UCLA and Oxford shoulder scores. Radiographic data included the change in the coracoclavicular distance on pre-operative, post-operative, and final follow-up plain radiographs.

## Results

### Study characteristics and clinical outcomes

Of the included studies, six-level II studies, five-level III studies, and six-level IV studies. This assessment was based on recommendations laid out in the Journal of Bone and Joint Surgery [17]. The surgical treatment used, patient demographics, clinical and radiological outcomes, complication rates, and methodology of each study are summarized in Table 1. The summary of the different available surgical techniques included in the above study is provided in Table 2.

**Table 2:** Summary of the different surgical techniques used.

Technique	Benefits	Drawbacks	Complications
Arthroscopic CC ligament reconstruction using synthetic graft	Higher patient satisfaction rates noted in some studies.	Similar loss to CC distance post operatively to open techniques. Similar post-operative outcome scores to open techniques.	Similar to open techniques, including cases of coracoid fracture, AC joint subluxation, loss of CC reduction, graft soft tissue reaction and implant failure.
Hook plate fixation	Very good CC distance reduction, similar post-operative outcome scores to reconstruction techniques. No cases of AC sublaxation.	Necessity for metalwork removal, increase of the CC distance following removal,	Cases of subacromial acrolysis, acromion erosion, and subacromial impingement. Occasionally periarticular ossification and scapular dyskinesis noted.
CC ligament reconstruction using synthetic implant (open technique)	Similar post-operative outcome scores to other techniques. Similar reduction of the CC distance to other techniques.	Similar loss to CC distance post-operatively to other techniques.	Soft tissue reaction, implant failure, coracoid fracture, loss of CC reduction, AC joint sublaxation.
Mini open AC repair (MINAR)/synthetic graft	Similar post-operative outcome scores to other techniques.	Loss of CC distance? (not evaluated)	Cases of periarticular ossification noted in the literature.
CC ligament reconstruction using autograft or allograft	Similar post-operative outcome scores to other techniques, similar reduction of the CC distance to other techniques.	Similar loss of the CC distance in comparison to hook plate and synthetic CC reconstruction techniques.	Similar to other techniques and occasionally pain and swelling at the site of harvesting (Autograft). Wound infection has been noted in the Allograft group.
Combined techniques	Similar post-operative outcome scores to other techniques, similar reduction of the CC distance to other techniques.	Increased operation time and cost? Loss of CC distance? (not evaluated)	Similar to previous techniques. No specific complication noted for the combined group.
Weaver-Dunn procedure	Similar post-operative outcome scores to other techniques.	Higher rate of loss of CC distance post-operatively in comparison to other techniques.	Higher rate of loss of CC distance post-operatively in comparison to other techniques.

### Choice of surgical procedure

#### Open versus arthroscopic surgery

Five studies were identified comparing the results of open and arthroscopic techniques, indicating that arthroscopic techniques have not proved any significant benefit to open techniques. The studies by Natera-Cisneros et al. [18] and Vrgoč et al. [12] compared the results of arthroscopic CC fixation with the hook plate and K-wires plus FiberTape (Arthrex Inc, Naples, Florida), respectively. In the former, the authors showed that patients who underwent the arthroscopic procedure experienced a higher global satisfaction rate and lower post-operative VAS score over the two-year follow-up period. In the latter, the authors did not find a statistically significant difference in outcomes between the open and arthroscopic procedures.

Li et al. compared the arthroscopic CC reconstruction with the modified Weaver-Dunn procedure and also found no statistically significant difference in American Shoulder and Elbow Surgeons (ASES) Shoulder Score and University of California Los Angeles (UCLA) Shoulder Score but did note that the loss of reduction of the CC distance was significantly lower after arthroscopic intervention [19].

Faggiani et al. compared the mini-open ACJ repair using the Minimal Invasive ACJ Reconstruction System (MINAR, Karl Storz, Tuttlingen, Germany) and the arthroscopic Dog Bone (Arthrex Inc, Naples, Florida) and also found no difference in clinical outcome scores [20].

A study by Stein et al. compared hook plate fixation with the arthroscopic double endobutton (Arthrex Inc, Naples, Florida) technique, they found improved post-operative Constant-Murley scores (95.3 vs. 90.2) after arthroscopic treatment. They also showed equivalent radiologic outcomes in terms of loss of CC reduction in the two techniques [21].

#### Acromioclavicular joint fixation with hook plate versus ligament reconstruction

Three studies in this review compared the results between the two different surgical approaches in question, with the hook plate fixation providing similar post-operative outcome scores to the ligament reconstruction techniques but with the necessity of a second operation for metalwork removal.

Fixation aims to restore the CC distance to allow healing of the injured ligaments. Several techniques focus on rigid and non-rigid fixation between the coracoid and the clavicle. Rigid fixation techniques include using K-wires or screws between the coracoid and clavicle and subsequent removal of the metalwork post-operatively. Non-rigid techniques have the advantage that they allow some movement and rotation of the clavicle while maintaining ACJ stability. Such techniques include the LockDown (Lockdown Medical, Minnesota, USA), formerly known as Surgilig, the modified Weaver-Dunn procedure, endobutton techniques, suture anchors, and the use of tendon allograft to reconstruct the CC (and sometimes also AC) ligaments.

Yoon et al. compared the results of hook plate fixation and synthetic CCL reconstruction and found no statistically significant difference in Constant-Murley or VAS scores of the patients in either group [22]. However, they did show that patients treated with a hook plate had a significantly larger reduction in the post-operative CC distance. Wang et al. also found no significant difference in the post-operative outcome scores of patients who underwent either a CC or AC tendon allograft reconstruction and patients who underwent hook plate fixation (mean Constant-Murley score 94.4 and 93.8, respectively) [23]. Similar post-operative outcome scores are also shown by Metzlaff et al., with no significant differences in the Constant-Murley and Taft scores between patients undergoing MINAR or hook plate at the 32-month follow-up [24].

### Comparison between CCL reconstruction techniques

#### Anatomic versus non-anatomic CCL reconstruction

Anatomical coracoclavicular reconstruction may lead to a better functional and radiological outcome than non-anatomical reconstruction. Anatomic restoration of the CCLs can be performed using synthetic ligaments, suture anchors, autograft, and allogenic tendon graft. There is controversy with regard to what can be considered anatomic reconstruction. Wellmann et al. defined two points of fixation on both the clavicle and coracoid as anatomic [25]. However, multiple fixation points and drill holes may increase the risk of coracoid and clavicle fractures (4–11%) [26].

A study by Chernchujit et al. compared the outcomes of arthroscopic anatomic and non-anatomic techniques of CC reconstruction and found significantly higher Specific AC (SAC) and Nottingham shoulder scores in those treated with anatomic procedures [27]. Furthermore, in over half of the patients in the non-anatomic arm, lost CC reduction was noted, suggesting that the anatomic technique provides a better reduction.

#### Synthetic versus biological graft

Soft tissue reaction following synthetic graft use remains a well-described complication. Various synthetic materials have been used, including polytetrafluoroethylene (GoreTex), polyethylene terephthalate (LARS), Dacron, and the Surgilig (now known as LockDown). They may provide primary stability and induce healing through encouraging colonization by fibroblasts and are used more commonly in chronic ACJ reconstruction. Despite good outcomes, these synthetic ligaments can cause significant foreign body reactions, and caution should be exercised in their use [28].

#### Weaver-Dunn versus synthetic or biological grafts

With the advent of new synthetic and biological grafts, the Weaver-Dunn and modified Weaver-Dunn procedures are losing their role in managing ACJ stability.

The CA ligament has 25% strength as compared to CCLs. Biomechanically, the vector of transferred CA ligament does not represent the vector of native CCLs [29]. One-third of cases have persistent instability [30, 31]. The sacrifice of the CA ligament also leads to loss of static restraint against anterosuperior humeral head migration in cases with rotator cuff failure [14, 32, 33].

Three studies in this review compared the use of biological and synthetic grafts versus the Weaver-Dunn procedure. Kumar et al. compared the results of the modified Weaver-Dunn procedure and the Surgilig (LockDown Medical, Minnesota, USA) technique and found that patients treated with Surgilig benefited from higher post-operative Oxford shoulder scores than the Weaver-Dunn group [34].

Similarly, Li et al. compared the results of the Weaver-Dunn procedure with the arthroscopic CC reconstruction using synthetic ligament. The authors underline that patients of the former group experienced a significantly higher rate of loss of reduction, questioning the contribution of the synthetic ligament in the restoration of vertical stability [19].

Kocaoglu et al. compared CC reconstruction using autograft through the GraftRope system (Arthrex Inc, Naples, Florida) with the modified Weaver-Dunn procedure using the TightRope system (Arthrex inc, Naples, Florida) [35]. They found that the group treated with the autograft had superior ASES and Constant-Murley scores. However, loss of reduction occurred in both groups in the 45-month follow-up assessment and was significantly higher in the modified Weaver-Dunn group.

#### Autograft versus allograft

The use of autograft or allograft for the anatomic reconstruction of the CC and AC ligaments in acute ACJ dislocation has gained popularity recently, with no clear evidence for one over the other. In this review, one study compared allograft and autograft reconstruction. Yin F et al. [36] tried to identify if the additional use of autograft or allograft to the hook plate fixation led to superior outcomes. No statistically significant difference was noted either in the clinical outcome scores or in the radiographic appearance.

#### Comparing combined techniques

Several studies captured in this review have described combined techniques, with the model of restoring vertical and horizontal plane stability gaining popularity. Barth et al. compared two arthroscopic endobutton techniques and found that adding horizontal plane ACJ stabilization led to superior radiographic and clinical outcomes.

Yin J et al. demonstrated that additional CC reconstruction in patients with hook plate fixation (in their study using the conjoint tendon) led to higher CMS and ASES scores and lower VAS scores in their 20-month follow-up period. They also showed that in all the patients treated with a hook plate, an increase in the CC distance was noted after the removal of metalwork [37].

The contribution of the CC reconstruction was also demonstrated in the study by Chang et al., who compared the results of the hook plate with the hook plate plus tape augmentation of the CCLs. Similarly, the CC reconstruction patients had higher UCLA and ASES shoulder scores post-operatively and experienced less pain [38].

Tang et al. compared the results of the double Endobutton technique and a suture anchor technique, with no significant difference in outcomes between the two [39].

#### Complications

Complications such as superficial wound infection, skin irritation, and implant failure were noted in many studies with no obvious trends in any particular technique. Subacromial osteolysis is a complication associated with the use of the hook plate [37, 38], whereas coracoid fracture remains a complication of CC ligament reconstruction techniques [40]. In addition, Chang et al. found that patients treated with a hook plate alone had a higher occurrence of subacromial osteolysis than those who underwent a combined technique of hook plate with CCL reconstruction [38]. Yoon et al. reported acromial erosion occurring in 9 out of 24 patients treated with hook plates [22]. All authors underline the necessity for the metalwork to be removed in patients treated with hook plates, and this in itself carries the additional morbidity of a second procedure.

Implant failure has been mentioned by several authors in patients undergoing a reconstructive procedure [18, 34, 41]. Natera-Cisneros et al. noted one case (out of 20) of implant failure after an arthroscopic CC fixation [18]. Barth et al. reported early loosening in 3 cases out of 105 treated with a double Endobutton technique [41]. These cases subsequently required further surgical stabilization. Overall, cases of implant failure were low, and in none of the studies reviewed was, the incidence particularly high.

Another recognized complication of CC reconstruction techniques is a coracoid fracture. Hou et al. [40] noted one case of coracoid fracture out of 11 patients undergoing semitendinosus tendon allograft with a single tunnel, while another case of coracoid fracture (in a series of 116 cases) was noted by Barth et al. [41] during double Endobutton arthroscopic technique. Heterotopic ossification is also noted by several authors, with Metzlaff et al. describing this phenomenon in 19 out of 44 patients [24] and Tang et al. finding this in 6 out of 56 [39].

## Discussion

This literature review provided an update on the surgical management of ACJ injuries, considering the evidence published since the last major literature review [15]. The recent comparative studies on arthroscopic versus open surgery have not proved any significant benefit to using the arthroscopic intervention. True rigid fixation techniques of CC stabilization techniques, such as the lag screw described by Bosworth [42], seem to have fallen out of favor, and the use of hook plate, while achieving a satisfactory reduction of the CC distance, carries the necessity of a second operation for metalwork removal. Non-anatomical CC ligament reconstruction techniques are becoming less popular, with modern anatomical CC reconstruction techniques focusing on both vertical and horizontal plane stability gaining popularity in the recent literature.

### Limitations

In the last major literature review, Modi et al. [15] commented that the recently published literature on the topic had been lacking in high-quality trials. Newer techniques have evolved over the last 8–10 years, but unfortunately, this trend has continued, with case series being the bulk of the new publications regarding ACJ injuries. There has continued to be a lack of homogeneity of the populations within each study with differing degrees of injury, the timing of intervention, and many different surgical techniques. Furthermore, numerous different outcomes scores and differing opinions on the use of radiological follow-up limit the external validity of each study. All these factors, together, make a drawing of meaningful conclusions on the optimal management difficult.

### Open versus arthroscopic surgery

Despite the increasing number of comparative studies, there is no clear evidence that arthroscopic intervention leads to superior results.

In the one study that compared arthroscopic CC reconstruction with hook plate, there was a slightly higher global satisfaction and improved VAS in the arthroscopic group [18]. The proponents of the arthroscopic technique argue about the 50% incidence of concomitant intra-articular glenohumeral pathologies associated with type III and V injuries, which could be diagnosed and treated simultaneously [14, 15]. Arthroscopic stabilization through ligament reconstruction also appears to produce good outcomes for posteriorly displaced distal clavicle fractures and acute dislocation, suggesting that arthroscopic intervention may have a role in treating concomitant injuries leading to ACJ instability.

### Rigid ACJ fixation (hook plate, K-wires) versus ligament reconstruction

One study in our reviewed series used a K-wire between the coracoid and clavicle in addition to a FibreTape. While rigid fixation methods provide more strength, the loss of motion increases the chances of loosening and breakage [18]. Furthermore, an additional procedure to remove metalwork is required. The principle of non-rigid methods allows some movement between the coracoid and clavicle while maintaining ACJ stability. As the biomechanics of the ACJ have become better understood, the importance of the vertical relationship between the coracoid and clavicle has been appreciated [41].

Fixation with a hook plate has been shown to reduce the post-operative CC distance better, however, it has also been shown that after removal of metalwork, the CC distance does increase again [37]. The hook plate does seem to produce good clinical outcomes, but there is some evidence that non-rigid ligament reconstruction techniques may give superior outcomes, as well as having the additional benefit of not requiring a further procedure to remove metalwork and avoid complications such as acromial erosion, subacromial osteolysis, and plate impingement [3].

### Comparison between CCL reconstruction techniques

Non-anatomical coracoclavicular reconstruction with the modified Weaver-Dunn is decreasingly popular. There is good outcome data reported in the case series, however, evidence suggests that anatomic reconstruction may result in superior functional outcomes [15]. This is likely due to the provision of both vertical and horizontal plane stability [41]. Indeed, there have been many recent publications comparing different methods of anatomic reconstruction, including the use of synthetic grafts, tendon autografts, and allografts. In one study comparing autograft and allograft, there was no difference in clinical or radiological outcomes [37]. Several studies have been published and described in the results section that compares different anatomic CCL reconstruction techniques – endobuttons, synthetic grafts, auto, and allograft. These are the most popular at present, based on recent literature. However, meaningful conclusions as to which of these are better are very difficult due to the heterogeneity between the studies and the lack of high-quality evidence. Recent evidence suggests that radiographic evaluation of the ACJ may not be reproducible, making comparing results between these studies difficult [16]. Patient factors such as gender-based differences in tunnel position and surgical factors such as time to surgery may contribute to the heterogeneity between studies of similar interventions [15, 16, 36, 43]. Furthermore, the complication profile presented in the results does not indicate a higher rate of failure of fixation in any particular technique.

In conclusion, despite the emerging literature on the reconstruction of the ACJ after injury, and evidence that several treatment options result in good outcomes, we cannot recommend the optimal treatment modality. This is due to the wide variety of options available, the heterogeneity between the studies on treatment options and methods to evaluate outcomes, and the lack of high-quality randomized studies in the literature.
